# Exploring steric sea level variability in the Eastern Tropical Atlantic Ocean: a three-decade study (1993–2022)

**DOI:** 10.1038/s41598-024-70862-0

**Published:** 2024-09-03

**Authors:** Franck Eitel Kemgang Ghomsi, Bayoumy Mohamed, Roshin P. Raj, Antonio Bonaduce, Babatunde J. Abiodun, Hazem Nagy, Graham D. Quartly, Ola M. Johannessen

**Affiliations:** 1 Deutsches Geodätisches Forschungsinstitut, Technische Universität München (DGFI-TUM), Munich, Germany; 2 Nansen-Tutu Center for Marine Environmental Research, Department of Oceanography, University of Cape Town, Cape Town, South Africa; 3 Geodesy Research Laboratory, National Institute of Cartography, P.O. Box 157, Yaoundé, Cameroon; 4 GeoHydrodynamics and Environment Research (GHER), University of Liège, Liège, Belgium; 5 Oceanography Department, Faculty of Science, Alexandria University, Alexandria, Egypt; 6 Nansen Environmental and Remote Sensing Center and Bjerknes Center for Climate Research, Bergen, Norway; 7 Marine Institute, Oranmore, Co.Galway, H91 R673 Ireland; 8 Plymouth Marine Laboratory, Plymouth, UK; 9 Nansen Scientific Society, Bergen, Norway

**Keywords:** Eastern Tropical Atlantic Ocean, Altimetry, Reanalysis, Sea level rise, Climate indices, Environmental impact, Geophysics, Physical oceanography, Natural hazards, Climate change, Climate and Earth system modelling, Climate-change impacts, Climate-change adaptation, Climate-change impacts, Climate-change mitigation, Environmental impact, Sustainability

## Abstract

Sea level rise (SLR) poses a significant threat to coastal regions worldwide, particularly affecting over 60 million people living below 10 m above sea level along the African coast. This study analyzes the spatio-temporal trends of sea level anomaly (SLA) and its components (thermosteric, halosteric and ocean mass) in the Eastern Tropical Atlantic Ocean (ETAO) from 1993 to 2022. The SLA trend for the ETAO, derived from satellite altimetry, is 3.52 ± 0.47 mm/year, similar to the global average of 3.56 ± 0.67 mm/year. Of the three upwelling regions, the Gulf of Guinea (GoG) shows the highest regional trend of 3.42 ± 0.12 mm/year. Using the ARMORD3D dataset, a positive thermosteric sea level trend of 0.88 ± 0.04 mm/year is observed, particularly in the equatorial and southern Atlantic regions. The steric component drives the interannual SLA variability, while the ocean mass component dominates the long-term trends, as confirmed by the GRACE and GRACE-FO missions for 2002–2022. For those two decades, the total SLR from altimetry amounts to 3.80 ± 0.8 mm/year, whilst the steric component is reduced to only 0.19 ± 0.05 mm/year, leaving a residual increase in the ETAO of 3.69 ± 0.5 mm/year. The independent mass change from GRACE amounts to 2.78 ± 0.6 mm/year for this region, which just closes the sea level budget within present uncertainty levels. Spatial analysis of the steric components indicates a warming along the equatorial African coast including the GoG and a freshening near Angola. Strong correlations with regional climate factors, particularly the Tropical South Atlantic Index, highlight the influence of persistent climate modes. These findings underscore the urgent need for mitigation and adaptation strategies to SLR in the ETAO, especially for densely populated coastal communities.

## Introduction

Sea level rise (SLR) is one of the most important consequences of global warming since human-induced factors such as greenhouse gas emissions have accelerated the rate of SLR in recent times. Fluctuations in sea levels have occurred naturally throughout Earth's history, spanning glacial cycles and interglacial periods^1–3^. The current rate of SLR, averaging 3.56 ± 0.67 mm/year since 1993, is uneven due to several factors^4^. Regional variations in SLR occur due to local environmental conditions such as sterodynamic variability (changes in ocean circulation, temperature, and salinity), coastal geomorphology, and land subsidence^5,6^.

Accurate determination of sea level anomalies (SLA) is essential for understanding these variations. An altimeter measurement typically calculates SLA as^7,8^:

$${\text{SLA }} = {\text{ Altitude }} - {\text{ Range }} - {\text{ Geoid }} - {\text{ Instrumental}}\;{\text{corrections }} - {\text{ Atmospheric}}\;{\text{corrections }} - {\text{ Oceanic}}\;{\text{corrections}}$$

This equation allows SLA to represent variations in sea level that are in geostrophic balance.

The total SLA^8^ can be expressed as the sum of the steric sea level anomaly (SSLA), which reflects changes in sea-level thermal expansion and salinity, the contribution of ocean mass related to land water storage, glaciers and ice sheets, and changes in circulation:

$${\text{SLA }} = {\text{ SSLA }} + {\text{ Ocean}}\;{\text{Mass}}\;{\text{Component }} + {\text{ changes}}\;{\text{in}}\;{\text{circulation}}$$

Ocean dynamics, including wind-driven currents and thermal expansion, induce mass redistribution, leading to uneven SLR^9^. Regional climate cycles, land water storage changes, and changes in ocean circulation and temperature further contribute to regional SLR variablity^10,11^.

**Fig. 1:**
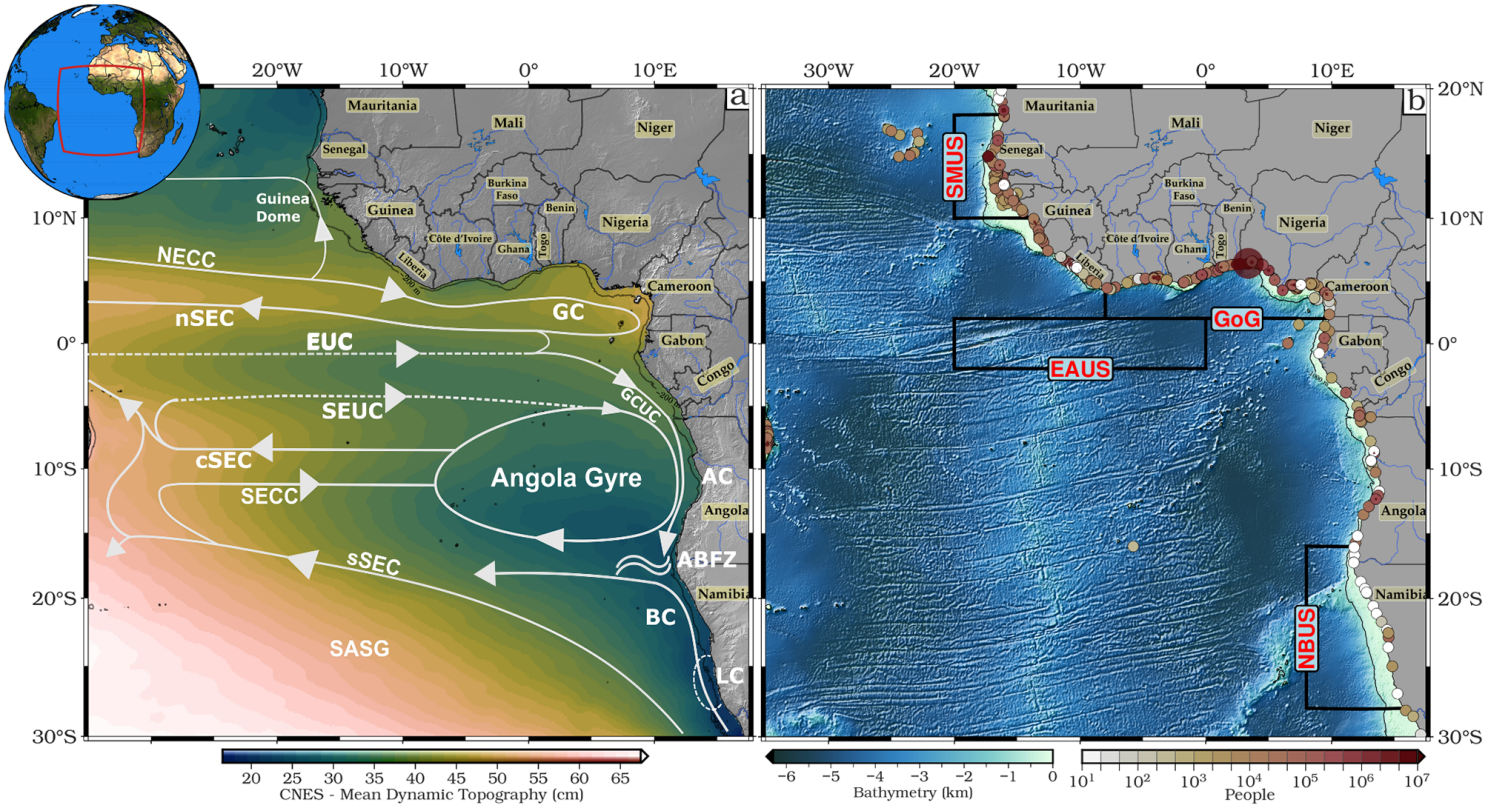
(**a**) Circulation scheme for the ETAO superimposed on the annual mean of the dynamic topography (MDT) for a 30-year period (1993–2022). Surface currents (solid arrows) and thermocline currents (dashed arrows) are depicted from Talley et al.^21^ and Marshall et al.^22^. The following currents are shown: the central, northern, and southern branches of the Southern Equatorial Current (cSEC, nSEC, and sSEC), the Gabon-Congo Undercurrent (GCUC), the Guinea Current (GC), the North Equatorial Countercurrent (NECC), the North Equatorial Undercurrent (NEUC), the South Equatorial Countercurrent (SECC), the South Equatorial Undercurrent (SEUC), and the Angola Current (AC). Additionally displayed are the Lüderitz cell (LC), the Guinea Dome, the Benguela Current (BC), the Angola Gyre, and the Angola-Benguela Frontal Zone (ABFZ). (**b**) shows the population of cities that are less than 10 m above sea level (from Nicholls et al.^23^) on a logarithmic scale over the ETAO, with the Gulf of Guinea (GoG, 8°W, 12°E; 2°N, 7°N) as the most exposed region. The acronyms EAUS, NBUS (20°W, 12°W; 10°N, 18°N) and SMUS (8°E, 16°E; 16°S, 28°S) stand for the Equatorial Atlantic Upwelling System, Namibian-Benguela Upwelling and the Senegal–Mauritania Upwelling respectively. The inset shows the position of the ETAO region along the tropical Atlantic.

The impacts of SLR on coastal communities and ecosystems are widespread^12,13^. These impacts include increased coastal flooding, erosion, and intrusion of saltwater into freshwater sources^10^. Low-lying island states and densely populated coastal areas, particularly in the Eastern Tropical Atlantic Ocean (ETAO: 30°S–20°N; 40°W–20°E, Fig. 1), which includes the Gulf of Guinea (GoG), the Namibia-Benguela upwelling system (NBUS), and the Senegal-Mauritania upwelling system (SMUS), are particularly vulnerable, with over 60 million people living less than 10 m above sea level facing significant risks of displacement and land loss^14,15^. In this context, understanding the drivers of SLR in the ETAO is crucial given its impacts on coastal communities and exacerbation by climate change.

Over the satellite era (since 1993), many previous studies have focused on the sea level change in the ETAO and its different sub-basins^16–19^. Dieng et al.^16^ analyzed SLAs along the West African coast from 2008 to 2014, highlighting that low resolution data did not capture small scale processes, leading to discrepancies in the representativeness of the Benguela system during geophysical corrections.

Ayinde et al.^17^ investigated interannual sea level variability in the GoG from 1993 to 2020 and found a consistent increase in SLA influenced by ocean heat content and large-scale atmospheric phenomena. Ghomsi et al.^19^ observed a mean SLR of 3.47 ± 0.10 mm/year in the equatorial region and 3.89 ± 0.10 mm/year along the coastal areas of the GoG, while emphasizing the role of coastal trapped Kelvin waves. However, they did not pay attention to the role of SLR’s mass component. Recent studies, such as that of Chen et al.^20^, have underlined the impact of Atlantic Niño. The Atlantic Niño is characterized by warm sea surface temperature (SST) anomalies in the eastern equatorial ocean basin and weaker than average trade winds throughout the east-central equatorial Atlantic. It influences the tropical Atlantic sea surface salinity (SSS), and is particularly associated with significant boreal summer freshening and atmospherically influenced winter meridional dipole patterns. However, further investigation into the relationship between climate variability and SLR fluctuations is warranted for a more comprehensive understanding of regional sea level variability.

Sea level dynamics in the ETAO are significantly influenced by large-scale forcing factors (see Table 1) such as the El Niño-Southern Oscillation (ENSO) and the Indian Ocean Dipole (IOD), although their regional importance differs^24–26^. ENSO, indicated by the Niño3 index, drives global sea level fluctuations. During El Niño events, warm water shifts from the Western Pacific to the Central Pacific, causing rainfall to enter the ocean rather than land, contributing approximately 7.62 mm to global sea level rise from 2022 to 2023^27^. The IOD affects SST differences in the Indian Ocean, altering regional weather patterns and ocean temperatures, which can impact sea levels through changes in circulation and thermal expansion. The Atlantic Multidecadal Oscillation (AMO) modifies SST in the North Atlantic, influencing ocean circulation and long-term sea level trends. Additionally, the Tropical Atlantic SST index (TASI) and the Tropical Northern and Southern Atlantic (TNA, TSA) indices reflect regional SST variations that affect sea levels through changes in circulation and thermal expansion. Together, these large-scale forcing factors drive changes in ocean temperature and water mass distribution, contributing to rising sea levels in the ETAO. Dièye et al.^18^ investigated the role of different climate modes on the SLA variability along the West African coast between 1993 and 2018. The authors found a significant correlation between the SLA interannual variability and the North Atlantic Oscillation (NAO), as well as the TNA index.

**Table 1:** Key large scale forcing factors of the regional climate system and their impact on oceanic and atmospheric patterns.

Climate index	Abbrev	Parameter	Location of high	Location of low	References	Effect
Atlantic multidecadal oscillation	AMO	North atlantic SST anomaly	Warmer waters in the North Atlantic	Cooler waters in the north atlantic	Enfield et al.^28^	Influences Atlantic SSTs and climate variability over multidecadal timescales
Indian ocean dipole mode index	DMI	Western—eastern tropical Indian ocean SST gradient	Positive phase: Warmer waters in the west, cooler waters in the east	Negative phase: Reversed temperature gradient	Saji et al.^29^; Liu et al.^30^	Affects monsoon patterns, rainfall in the Indian Ocean region (East Africa, Australia, and Southeast Asia.)
Niño3 index	Niño3 (150°W–90°W, 5°S–5°N)	Eastern tropical pacific SST anomaly	Eastern pacific	Eastern pacific	Trenberth and Stepaniak^31^	Key indicator of El Niño-Southern Oscillation (ENSO) events, affecting global atmospheric circulation and regional climates
Tropical atlantic SST index	TASI	Gradient of meridional SST anomalies in the tropical Atlantic	Tropical atlantic	Tropical atlantic	Chang et al.^32^	Influences Atlantic hurricane activity and West African monsoon. It is associated with a potential decadal 'dipole' mode of coupled variability in the tropical Atlantic
Tropical northern atlantic index	TNA (55°W–15°W, 5°N–25°N)	SST anomalies in the northern tropical atlantic ocean	Northern tropical atlantic	Northern tropical atlantic	Enfield et al.^28^	Affects Atlantic hurricane activity and rainfall over the Americas
Tropical southern atlantic index	TSA (30°W–10°E, 20°S–Eq)	SST anomalies in the southern tropical atlantic ocean	Southern tropical atlantic	Southern tropical atlantic	Enfield et al.^28^	Indicator of the surface temperatures in the GoG, the eastern tropical South Atlantic Ocean. Influences rainfall patterns in South America and Africa
Atlantic niño	ATL3 or EAUS (3°N–3°S, 0–20°W)	Eastern tropical atlantic SST anomaly	Eastern tropical atlantic	Eastern tropical atlantic	Chen et al.^20^; Liu et al.^30^	Influences West African summer monsoon, leading to reduced rainfall in the Sahel region and increased flooding in northeastern South America and the West African sub-Sahel countries bordering the GoG

Despite recent advances, some research gaps remain, thereby limiting our understanding of SLR over the ETAO. For instance, while previous studies have examined SLAs, the sea level variability, and its budget in the ETAO, the specific role of the mass component has not been thoroughly quantified. Additionally, existing research does not cover the latest trends and potential accelerations in SLR, which is necessary for providing an updated perspective on sea level changes. Likewise, the influence of large-scale climate factors on SLR in the ETAO has not been elucidated, which could provide deeper insights into regional sea level variability.

This study therefore aims to quantify the individual contributions of thermosteric, halosteric as well as ocean mass components to SLR across the ETAO. Our purpose is not only to extend the temporal analysis of SLR trends, but also to shed light on the links between large-scale forcing factors of the regional climate system on SLR. We investigate the interannual variability and trends of total SLA and the contributions of thermosteric and halosteric effects in the ETAO. To achieve this, we use altimetry and temperature and salinity profiles obtained from ARMOR3D^33^ from 1993 to 2022. ARMOR3D is a multi-decadal product blending data from satellites and in situ sensors (ship and Argo floats). It ensures the accuracy of salinity data by effectively managing salinity drift through a rigorous process of real-time quality control and delayed-mode adjustments^34,35^. In our study, we estimate the relative contributions of the atmospheric, glacial isostatic adjustment (GIA), thermosteric, halosteric and total steric effects on the SLA variability and trends. Then, we examine temporal trends in SLAs and their components. Finally, we examine interannual variability of SLA and the role of large-scale forcing factors on sea level change.

## Study area

The ETAO showcases distinctive features in its circulation patterns (Fig. 1). Encompassing the region between 3°N to 3°S and from 20°W to 0°W, the EAUS governs the open ocean dynamics within this area. Moreover, the ETAO exhibits the presence of two prominent coastal upwelling regions, further contributing to its dynamics. The GoG Upwelling System extends from 8°W to 3°E, while the Tropical Angolan Upwelling System stretches from 6° to 17°S along a similar coastal band. The Guinea Current (GC), which is at its strongest during the boreal summer, is nourished by the NECC as it travels eastwards through the ETAO^36^ and can be regarded as its eastern extension. The GC is linked to the Canary Current that flows southward down the coast of Africa, and the position of the Intertropical Convergence Zone varies with the seasons, which impacts this connection. The GC exhibits a year-round eastward flow, reaching its highest mean velocity of 42 cm/s at 8°W, 4°N^37^. The Angola Current (AC) is a fast-moving, narrow current that flows along the Angolan coast south of the GC. It is shaped by equatorial Atlantic variability and transports saline and warm water near to the coast^38,39^. The Angola Gyre, an area of cooler waters linked to the cyclonic transition of the South Equatorial Undercurrent (SEUC), lies close to the AC^40^. The Angola Gyre is bounded by the South Equatorial Counter Current (sSEC), SEUC and AC, and stands as a permanent upwelling feature embedded within the large-scale cyclonic South Atlantic Tropical Gyre. It connects the South Equatorial Countercurrent (SECC) to the AC via a cyclonic gyre centered at 10°S and 9°E^41^. Bakun^42^ discovered a correlation between local cooling and the intensification of the GC, which distributes fresh water from the African coast to the basin's interior, north of the GoG. The thermocline waters are transported eastward by the Equatorial Undercurrent (EUC) at the equator, in the direction of the EAUS. These waters are nearly exclusively of southern hemisphere origin because of the Atlantic Meridional Overturning Circulation^43–45^. Some of the water returns to resupply the southward-flowing Gabon-Congo Undercurrent (GCUC) and the AC, and some recirculates into the westward branches of the South Equatorial Current (SEC), the Central and the Northern South Equatorial Currents (cSEC, nSEC)^46,47^.

## Results and discussion

### Spatial and temporal trends of SLAs and its components

**Fig. 2:**
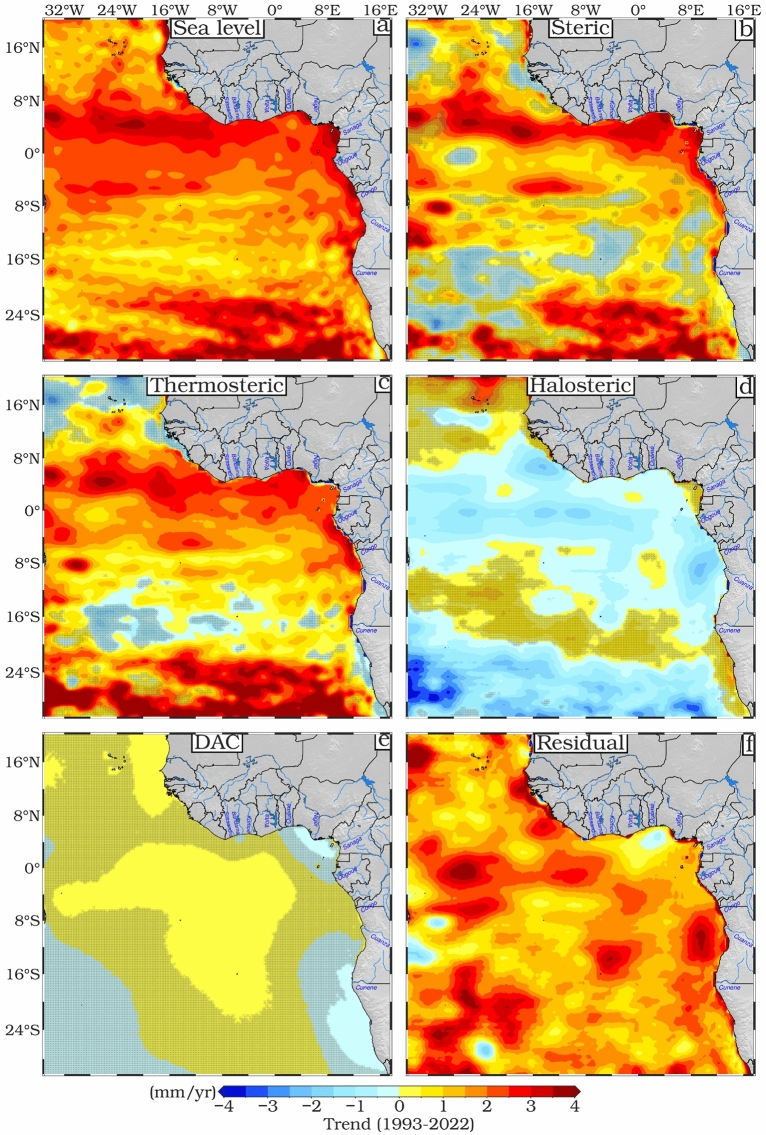
Spatial patterns of trends (mm/year) from 1993 to 2022 for (**a**) SLA from altimetry after the GIA correction, (**b**) steric effect above 700 m from ARMOR3D after the GIA correction, (**c**) GIA-corrected thermosteric effect above 700 m from ARMOR3D, (**d**) GIA-corrected halosteric effect above 700 m from ARMOR3D; (**e**) DAC and (**f**) altimetry after the steric component and the GIA correction were taken out (residual). Mean and seasonal cycles were removed from all-time series at each grid point. Regions with trends not statistically significant at the 95% confidence interval are hatched.

The spatial distribution of the overall sea level trend and its components, including residual sea level (total sea level minus the steric component, which represents the mass contribution), is shown in Fig. 2, and the trends in key marine ecosystems are summarized in Table 2. The total SLA shows positive and significant (*p* < 0.05) trends throughout the ETAO (Fig. 2a). The spatial pattern of SLA increase is uneven, with stronger local trends in the northern and southern areas. These trends are predominantly in the range of 1–4 mm/year (Fig. 2a). The highest SLA trend values (up to 4 mm/year) were found between 0° and 8°N, which coincides with the upwelling areas covered by the NECC, the nSEC and the GoG coastal upwelling area (see Fig. 1 for locations). In addition, the region of high positive trend extends along the entire coast from Senegal to the vicinity of Angola. Higher trend values are also found in the Southern part of the domain, mostly within the anticyclonic South Atlantic Subtropical Gyre (SASG). The SASG influences sea level mainly through its oceanic circulation, which is driven by wind stress curl and extends roughly between 55°W–10°E and 45°S–15°S^48^. The lowest SLA trend values (< 1 mm/year) are found near the location of the cyclonic Angola Gyre (between 8° and 20°S), as well as off the wind-driven upwelling region across the Namibian shelf (Fig. 2a). The GIA correction to the altimetry data (dGeoid) is negative throughout the region, with values ranging from about − 0.25 to − 0.30 mm/year^49^.

**Table 2:** Trends in the mean sea level and its related components over the large marine regions of the ETAO from 1993 to 2022 as shown in Fig. 1b.

Region	Sea level (mm/year)	Steric (mm/year)	Thermosteric (mm/year)	Halosteric (mm/year)	Residual (mm/year)	DAC (mm/year)
Gulf of Guinea (GoG)	3.42 ± 0.12	1.00 ± 0.10	1.30 ± 0.10	− 0.27 ± 0.03	2.58 ± 0.07	0.05 ± 0.03
Senegalo–Mauritanian Upwelling System (SMUS)	2.85 ± 0.10	0.36 ± 0.09	0.34 ± 0.11	− 0.08 ± 0.04	2.59 ± 0.08	0.05 ± 0.03
Namibian-Benguela Upwelling System (NBUS)	3.18 ± 0.06	0.57 ± 0.06	0.76 ± 0.07	− 0.22 ± 0.03	2.71 ± 0.06	0.06 ± 0.04

Figure 2c,d show the individual contributions of temperature and salinity expansion and contraction to the total SLA. Almost the entire region has positive thermosteric rates of sea level change (Fig. 2c). Importantly, regions with pronounced SLA increase (Fig. 2b) are aligned with areas showing prominent steric sea level changes, which are predominantly driven by the thermosteric variability illustrated in Fig. 2c. These results confirm that, for our region, the steric sea level is primarily dominated by its thermosteric component. The average spatial trends in SLA (Fig. 2a), steric (Fig. 2b) and thermosteric (Fig. 2c) components were 3.48, 0.55 and 0.86 mm/year, respectively, over the whole study period. The halosteric contribution to the trend is not statistically significant (*p* < 0.05) throughout the study area and contributes negatively to the overall steric effect with a mean trend of − 0.36 mm/year. The highest negative halosteric trend values were found in the SASG, mostly in the southwestern region, which has the largest salinity-driven sea level fall. In most places, however, the halosteric trend (Fig. 2d) sea level is negative, moderating the thermosteric rise in sea level. The pattern of the halosteric trend shows an opposite variation to the thermosteric trend (Fig. 2c), a similar contrast between halosteric and thermosteric trends is observed in the North Atlantic, albeit for different reasons. This could be due to the increase in the thermosteric contribution near the equator^50^, which is associated with a high evaporation rate, leading to an increase in salinity and thus a decrease in sea level trend in this region due to halosteric contributions.

To account for the atmospheric contribution to the sea level trend, the Dynamic Atmospheric Correction (DAC) is applied. It is an important correction, adjusting for the effects of atmospheric pressure and wind on the ocean's surface^51^. It ensures that the satellite-derived sea level data reflect true oceanic changes rather than atmospheric fluctuations. DAC integrates low-frequency data from the inverted barometer correction with outputs from the barotropic MOG2D-G model^52,53^, improving the representation of high-frequency atmospheric forcing. However, for our region, the overall trend in DAC is small, with even parts deemed significant (*p* < 0.05) being of order ± 0.05 mm/year. The residual SLA components (i.e., the total SLA minus GIA and the total steric) shows a significant (*p* < 0.05) contribution and trend over the whole study area (Fig. 2f). These results suggest that the mass component (i.e., the SLA residual) is the dominant contributor to the SLA trend in ETAO.

Table 2 shows the trends in SLAs and their associated components and atmospheric drivers across the ETAO sub-regions. The GoG shows the highest sea level trend of 3.42 ± 0.12 mm/year, driven by significant contributions from both the steric (1.00 ± 0.10 mm/year) and residual (2.58 ± 0.07 mm/year) components. The thermosteric component, reflecting the warming, has a particular influence on this region, contributing 1.30 ± 0.10 mm/year to sea level rise. This is consistent with the findings of Cardoso et al.^54^, who identified higher sea level trends ranging from 3.5 to 4 mm/year south of 10°N, influenced by the NECC. In our study, the three upwelling regions (GoG, SMUS and NBUS) show a range of values for total SLR (3.42, 2.85 and 3.18 mm/year respectively), but their residuals after removal of steric components are quite similar (see Table 2). This similarity suggests that the observed SLR is primarily associated with mass changes, likely driven by the reduction of land ice and emptying of aquifers, whose effects are nearly uniform across these regions. The trend in DAC is almost uniform across these regions and is minimal. Thus, it is the steric components, especially the thermosteric, which are crucial in explaining spatial differences in SLR and associated interannual variability.

The analysis with EN4_g10 (an alternative temperature and salinity dataset, see Gouretski and Reseghetti^55^; Good et al.^56^) finds similar patterns (Fig. S1 in Supporting Information), with a banded structure to the thermosteric component, a small negative halosteric one and a broadly uniform residual representing the mass contribution. However, with EN4_g10, the trend in steric SLR is somewhat larger (1.07 mm/year compared with 0.586 mm/year for ARMOR3D) and consequently the inferred trend in mass contribution is reduced (Fig. S2 in Supporting Information). Steric increase below the 700 m depth limit of ARMOR3D is expected to be minimal following Church et al.^57^, who estimated the typical Steric changes between 700 and 3000 m to be less than 0.1 mm/year. While we acknowledge the potential for minor errors in the steric component, the consistency of the datasets reinforces our conclusion that the residual sea level of the region primarily reflects changes in ocean mass. This suggests significant contributions to the observed residual component, such as changes in ocean circulation, wind stress, and variations in land water storage. In addition to these local influences, global factors such as ice sheet and glacier melt also may contribute to the observed residual component. Furthermore, freshwater input from major river systems in the region may play a role in the observed changes. Conversely, the steric components, especially the thermosteric, are crucial in explaining interannual variability. This is supported by a strong correlation (r = 0.79) between the steric interannual variance and the total interannual variance (see Fig. S3 in Supporting Information), indicating that the steric component accounts for a substantial portion of the interannual variability observed across the ETAO.

### Temperature and salinity changes

**Fig. 3:**
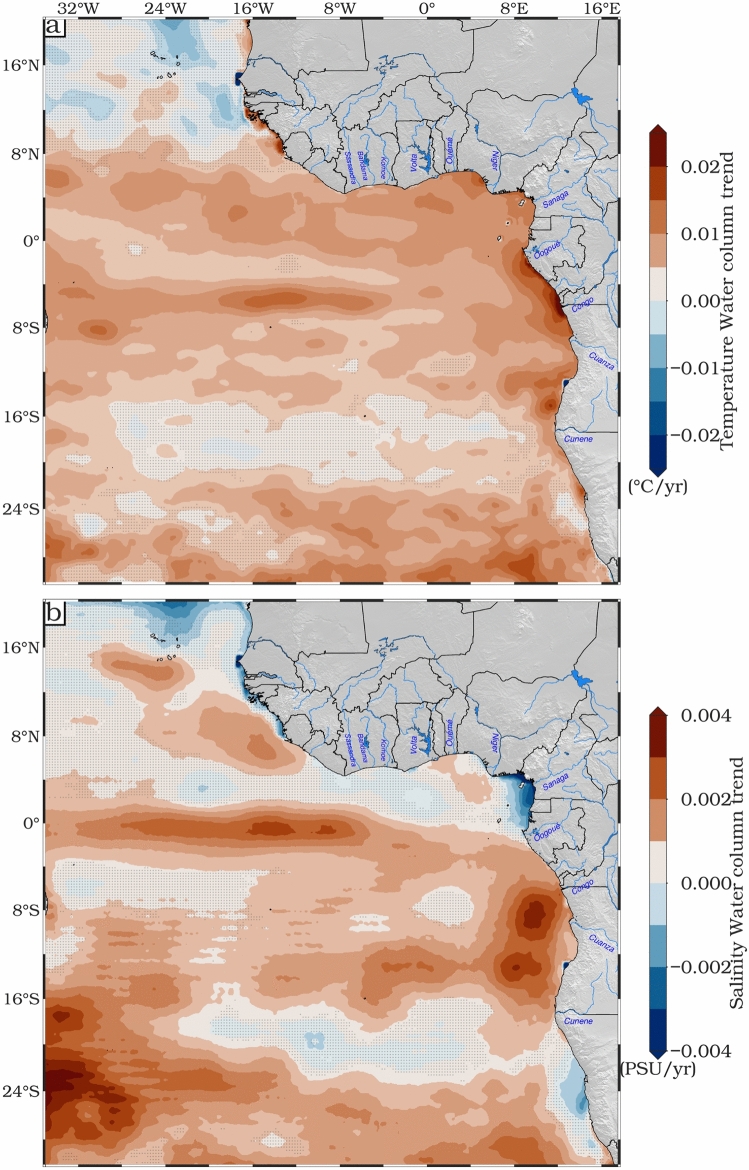
Spatial distribution trends (mm/year) of the average water column (from 0 to 700 m) (**a**) temperature and (**b**) salinity over the period 1993–2022. Regions where the trends are not statistically significant at the 95% confidence interval are stippled.

Figure 3 shows maps of the spatial trend of the averaged water column (0–700 m depth) temperature and salinity for the period 1993–2022, from the ARMOR3D dataset. High spatial variability of the mean water column temperature (Fig. 3a) is observed over the ETAO, ranging from − 0.01 to 0.03 °C/year. Statistically significant trends (*p* < 0.05) that exceed 0.02 °C/year are found between 8°N and 16°S. A pronounced positive temperature trend pattern is found along the equatorial region and towards the African coast of the ETAO. The most pronounced signature is observed along the Gabon-Angola coastal margin, where there are major outflows from the Ogooué and Congo rivers. However, this region is also marked by increased salinity, which, together, could indicate a reduced total volume of Congo river outflow^58^ into the Atlantic Ocean can affect ocean density and circulation dynamics, intensifying the warming of the water column. On the other hand, the water column temperature trend is not significant (*p* < 0.05) in two regions: the northern part of the ETAO (> 8°N) and between 16°S and 22°S. Within the anticyclonic SASG (in the southwestern corner of our region), there is convergence of warm surface waters towards the centre of the gyre^59^, which seems to have deepened the thermocline and warmed the subsurface water column, further contributing to the rise in steric sea level. These results are consistent with Fig. 2b. The non-significant water column temperature trend may be due to the frontal zone that defines the front of the Benguela Current to the northwest and the Angola Current to the south^60,61^. The spatial mean temperature trend over the whole domain is 5.5 × 10^–3^ °C/year.

The water column salinity trend is not significant over most of the domain during the study period (Fig. 3b). The strongest salinity trend (> 2 × 10^–3^ PSU/year) is observed in the southwestern part of the ETAO, which corresponds to the main corridor of Agulhas Rings^62^ bearing salty Indian Ocean water, the Angolan coast and along the equator. The salinity trend observed around Angola may be influenced by several factors. The reduced discharge of the Congo River^58^ is likely to be a major contributor to the increased salinity in this region. Along the Angolan coast, coastal upwelling processes bring deep, highly saline waters to the surface, further increasing salinity in the water column. In addition, mixing processes, influenced by factors such as wind stress and the presence of the Angola Gyre, play a role in redistributing salt within the water column, contributing to the observed salinity trend^63^. Salinity in this area is rising temporally as a result of the Angolan upwelling. Furthermore, ocean currents, such as the South Equatorial Current through its connection to the Angola Gyre, play a crucial role in transporting more saline water towards the coast, thereby amplifying the salinity trend. The increased salinity trend along the equatorial band may be linked to increasing evaporation rates that exceed precipitation levels in equatorial regions^64^. Additionally, the intensity and position of the Intertropical Convergence Zone (ITCZ) play a pivotal role in modulating salinity patterns. Shifts in the ITCZ can alter precipitation distribution and intensity, affecting the freshwater balance and consequently the salinity in the region. For instance, a more northerly position of the ITCZ can lead to reduced rainfall over the equatorial Atlantic, enhancing salinity through decreased freshwater input. A negative salinity trend is observed along the Namibian coasts, mostly marked along the NBUS (the Lüderitz cells which is one of the most prominent and intense upwelling cells within the Benguela Upwelling System and is located around 26°–27°S off the coast of Namibia) and off the Cameroon coastal margin. The observed decrease in salinity may indicate increased wind-induced upwelling, bringing fresher and colder waters to the surface.

Furthermore, reduced river outflow (such as from the Sanaga river of Cameroon) can also influence salinity trends. In addition, the interaction of ocean currents and mixing processes off the coast of Cameroon can contribute to the dilution of surface waters^65,66^, resulting in a negative salinity trend. In general, both temperature and salinity trends show the same pattern along the equatorial domain and the Gabon-Angola coast. Regions with non-significant trends in both temperature and salinity are likely influenced by the ABFZ, where the warm Angola Current interacts with the cold Benguela Current from the south.

**Fig. 4:**
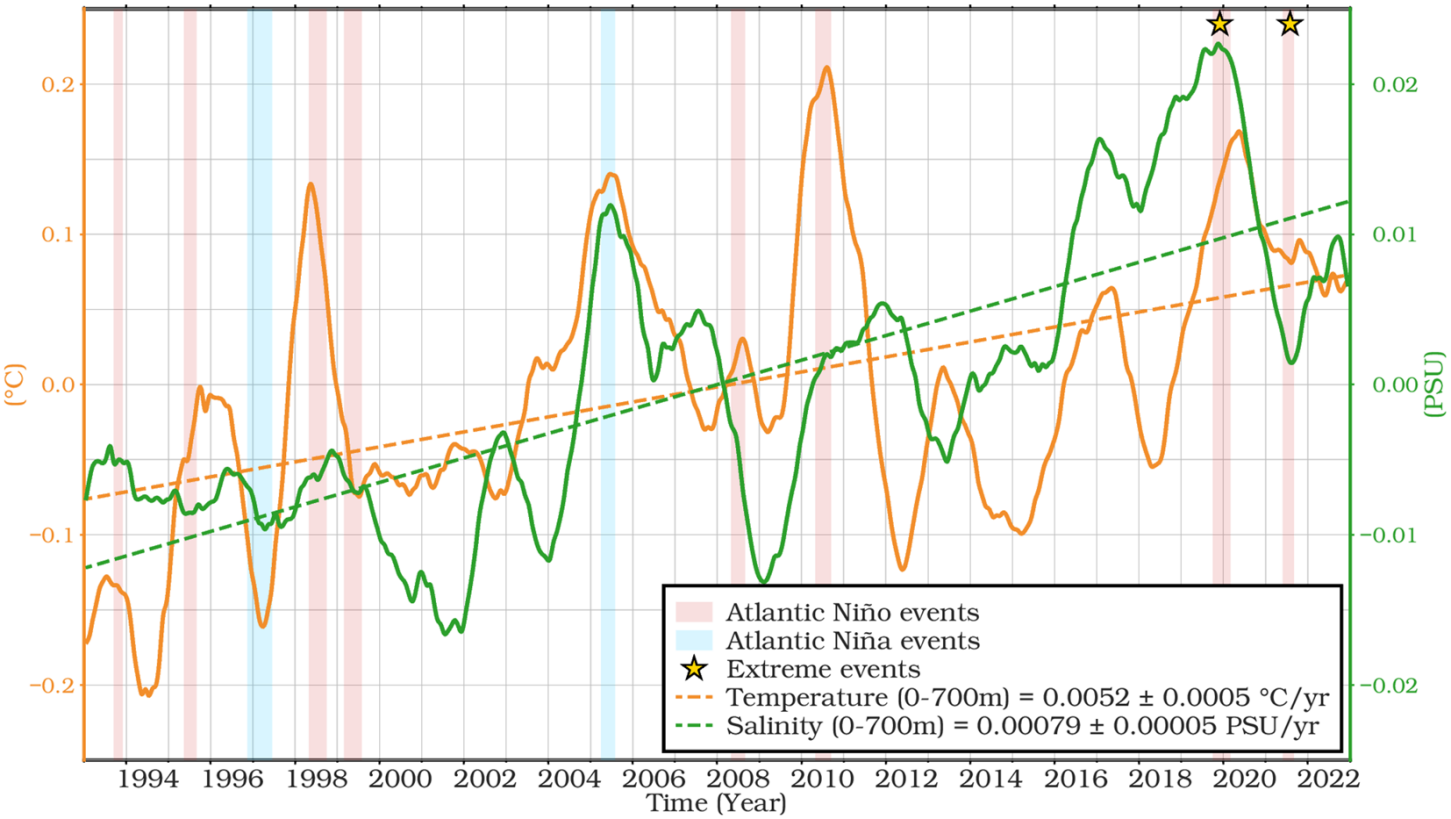
De-seasoned monthly time series and linear trends (mm/year) over the water column from 0–700 m of temperature and salinity from ARMOR3D over the ETAO for 1993–2022.

In the ETAO from 1993 to 2022, both averaged water column (0–700 m) temperature and salinity show a sustained upward trend (Fig. 4). This is reflected in the positive slopes of their linear trend lines.

Temperature variations show distinct peaks in certain years (Fig. 4), notably 1996, 1998, 2005, 2008, 2010 and late 2019 to early 2020. These peaks often coincide with Atlantic Niño events^19,67–71^, with the late 2019 episode standing out as particularly intense over the ETAO^17^. Similarly, salinity across the water column shows high variability, with an overall increase observed over time. Notably, similar peaks were found in salinity in 2005 and late 2019 to early 2020. The observed increase in ocean temperature is likely to have increased evaporation rates, leading to higher salt concentrations in surface waters and consequently, increased surface salinity^72,73^. In addition, changes in ocean circulation patterns, precipitation regimes, and river discharge all contribute to fluctuations in salinity. These insights are consistent with the report of the Intergovernmental Panel on Climate Change^74,75^, which highlights the link between rising oceanic global temperatures and increasing ocean salinity. Such changes in oceanic conditions have significant implications for sea level rise in the study area.

### Temporal trends analysis of SLAs and its components over the ETAO

**Fig. 5:**
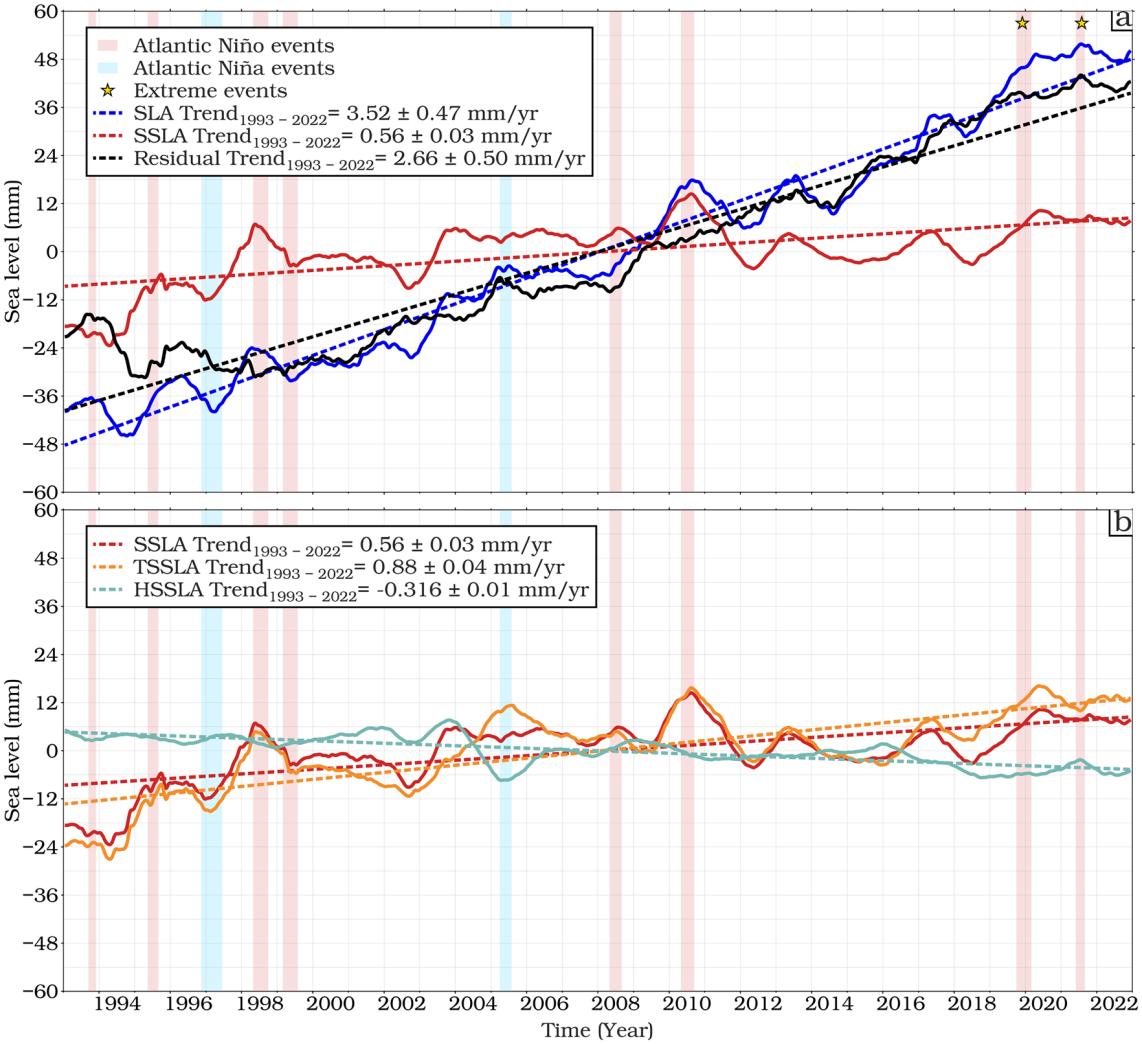
De-seasoned monthly time series and linear trends (mm/year) for mean (**a**) SLA, steric, and residual (SLA minus steric) components and (**b**) steric, thermosteric, and halosteric sea level components from 0 to 700 m from ARMOR3D over the ETAO for 1993–2022.

The temporal variability and the seasonal time series of the total SLA, thermosteric, halosteric and residual between 1993 and 2022 are shown in Fig. 5. All-time series have been low pass filtered with a 12-month cut-off period for comparison and to emphasize the interannual and long-term variability. Strong interannual variability is found in 2010 in all the components which are related to the 2010 Central Atlantic Niños^19,71,76^ and remote 2010 El Niño-Southern Oscillation (ENSO) events. The average temporal trends of the total, steric and residual SLAs are about 3.52 ± 0.47, 0.56 ± 0.03 and 2.66 ± 0.50 mm/year, respectively, over the whole study period (see Fig. 5a). Notably, the residual component (absolute sea level minus Steric Sea level), reflecting the mass contribution, has the largest effect on the sea level budget. The accelerated trend of SLAs observed between 2010 and 2021 could be related to the decadal oscillation over the decades. There have been four major warming extremes, with the late 2019 and boreal summer 2021 Atlantic Niños being the most intense of the last four decades which, appear to have been triggered by warming along the GoG^19^ and Angola-Namibian coast^68–70^.

The observed residual sea level trend mirrors the trend in sea level rise (Fig. 5a). The partitioning of the total steric of SLAs into two components, thermosteric and halosteric, is shown in Fig. 5b. The thermosteric component shows a significant positive trend of 0.88 ± 0.04 mm/year between 1993 and 2022, while the halosteric trend is weaker^77–79^, but still significant (*p* < 0.05) with − 0.32 ± 0.01 mm/year. A strong interannual variability is observed in the thermosteric components, with the strongest peaks in 1998, 2010 and 2020, coinciding with the strong phases of El Niño events, whereas the halosteric shows little response to these events.

The residual sea level rise of 2.66 mm/year principally relates to changes in ocean mass through melting of glaciers and land ice. Analysis of EN4_g10 shows a mean steric SLR of 1.07 mm/year and a mean residual of 2.15 mm/year (see Fig. S1 in Supporting Information). Independent estimates of the mass changes using GRACE data for 2004–2015 are in the range of 2.13 ± 0.14 mm/year^80^ and 1.56 mm/year^81^. These values are both for the barystatic component i.e. global means, whereas our Fig. 2f purports to show regional variations, termed "manometric", which include aspects related to changes in circulation.

**Fig. 6:**
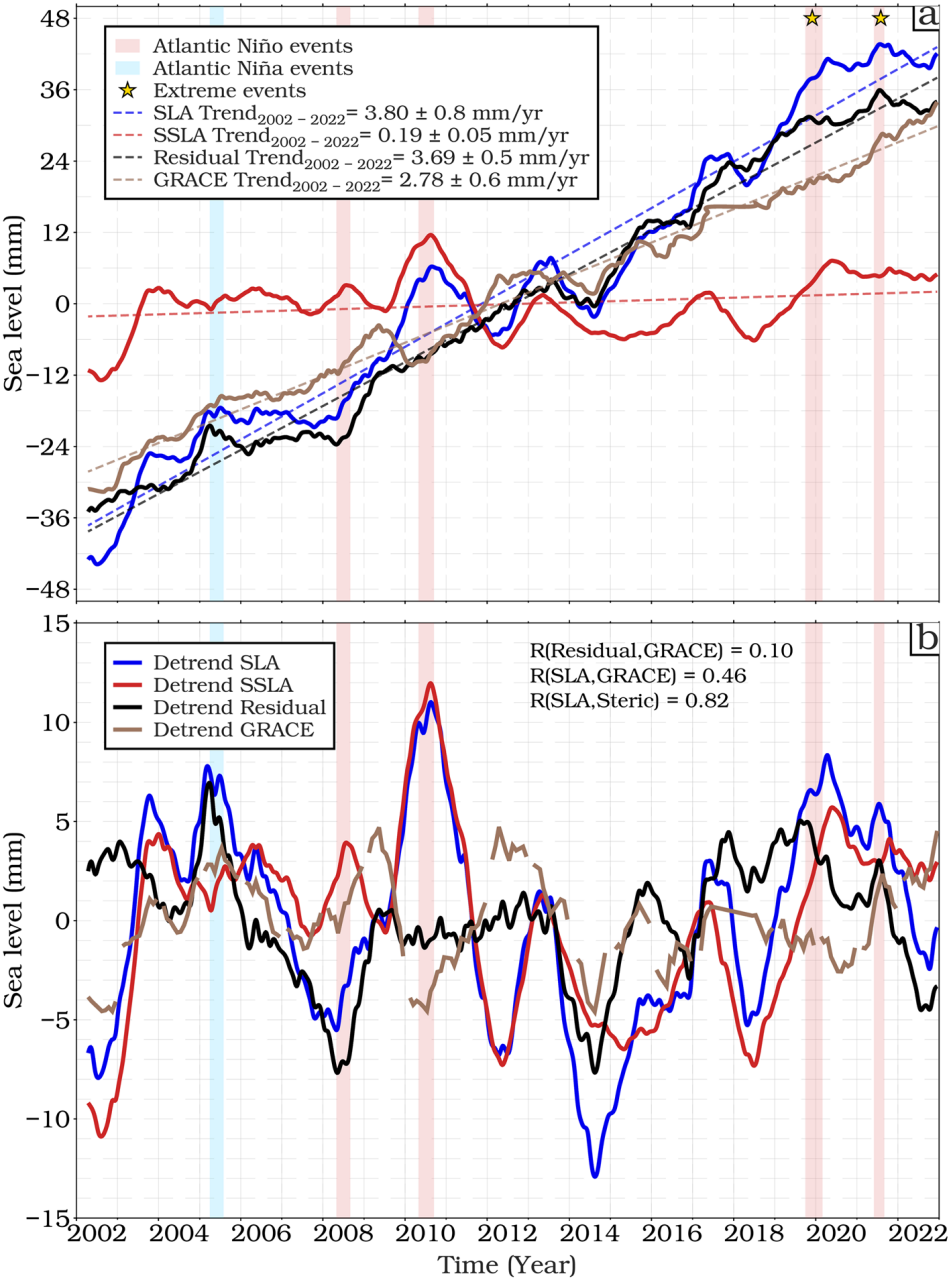
(**a**) Similar to Fig. 5, but lifetimes and baseline references between GRACE and altimetry missions (04/2002- 12/2022), adding the de-seasoned monthly time series and linear trends (mm/year) of the sea level mass component from GRACE/GRACE-FO, (**b**) Detrended monthly SLA, steric, residual (SLA minus steric) components and ocean mass component sea level (GRACE).

In Fig. 6 we focus on the period from April 2002 to December 2022, which corresponds to the availability of data from the Gravity Recovery and Climate Experiment (GRACE) and its follow-on mission (GRACE-FO). These timescales allow an extensive analysis of sea level dynamics and budgets. Over this reduced period, the rate of total SLR is greater (3.80 mm/year) but the steric contribution much reduced (0.19 mm/year), implying a residual of 3.69 mm/year. This exceeds the mass contribution derived from GRACE data (2.78 mm/year), although the uncertainty ranges for these trends do overlap. This near-closure suggests that the observed sea level change in the region can be largely explained by the combined effects of ocean mass changes and steric variations.

As noted by Llovel et al.^82^ more work is required to reduce these uncertainties before we can look closely at any discrepancy between these estimates. In addition, the coarse spatial and temporal sampling of GRACE compared to the higher resolution altimetry and ARMOR3D data may lead to aliasing effects when deriving the residual and an inability to capture small-scale or high-frequency mass redistribution processes contributing to sea level changes such as coastal currents, eddies, or local wind-driven processes^83^. Finally, uncertainties in the GRACE measurements themselves may arise from factors such as signal leakage, post-processing techniques, and background models used in the gravity field solutions^84^. The mass changes contribute to an almost uniform rise in sea level across the ETAO region, whereas the variability is more closely tied to steric adjustments, influenced by regional climate modes.

### Role of climate modes on sea level change

**Fig. 7:**
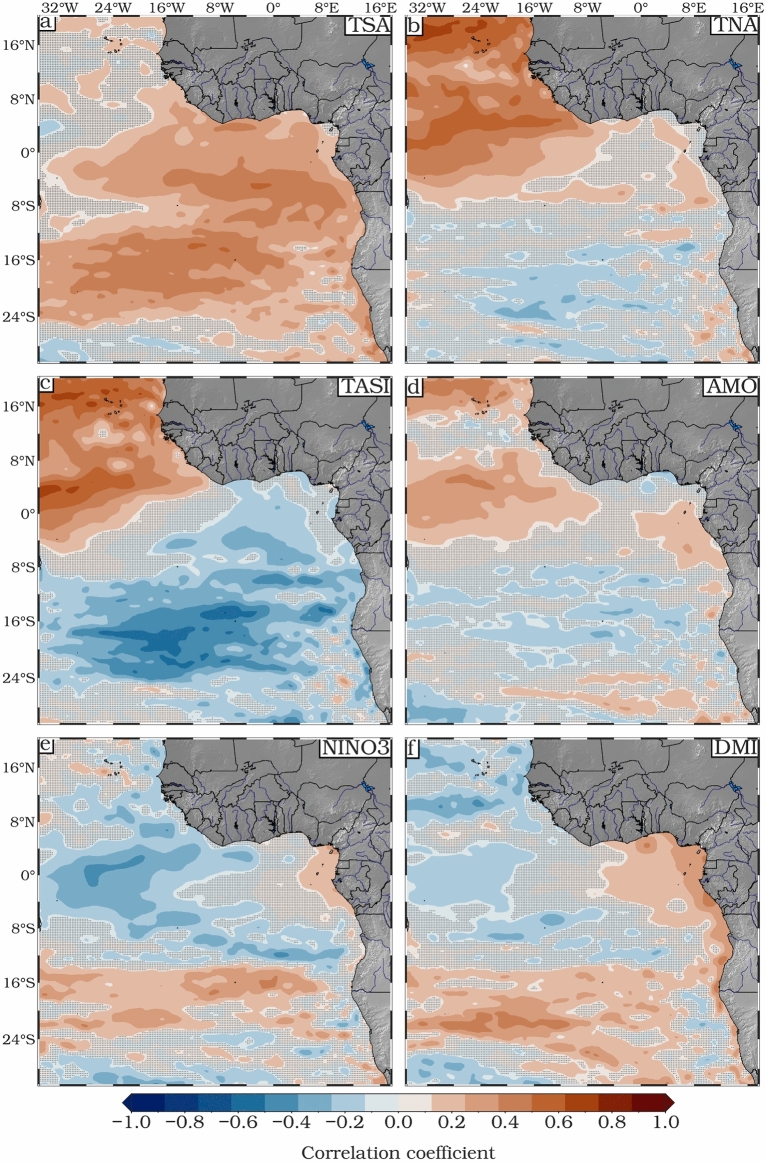
Spatial correlation maps between (**a**) TSA, (**b**) TNA, (**c**) TASI, (**d**) AMO, (**e**) Nino3, (**f**) DMI and monthly mean SLA for the period 1993–2022. Black dots indicate regions where the correlation is not significant.

In the following part we investigate the spatial correlation between SLAs and six climate indices (TSA, TNA, TASI, NIÑO3, DMI, and AMO). We have carefully selected these indices based on our analysis of correlations (Fig. 7), which show significant correlations with SLAs. Note, there is some similarity between some of these patterns (e.g., Fig. 7b,c) because the time series of these indices are not orthogonal.

In the South Atlantic region, we found a highly significant correlation between the SLAs and the TSA index (Fig. 7a). The TSA index serves as an important indicator of SLA variability, especially in regions such as the GoG and along the equatorial region. This importance was emphasized in studies by Utida et al.^85^, Sheng et al.^86^ and Nana et al.^87,88^. The TSA index captures the variability of the dominant features (i.e., the Atlantic cold air tongue and the permanent coastal upwelling along the GoG, which are pronounced off Côte d’Ivoire) that drive SLA variability within the ETAO. We found a positive correlation (r > 0.7) across most of the region, suggesting that the TSA index has a broad positive influence on SLAs in the region. The TNA shows a dipolar nature, with strong positive correlations in the northern part of the ETAO (up to 0.7), with weak insignificant correlations elsewhere (Fig. 7b). These results are consistent with those of Xie and Carton^89^, who found that TNA can influence the interhemispheric gradient of sea surface temperature in the tropical Atlantic, highlighting the far-reaching impact of these anomalies on climate dynamics. The correlation with the TASI index reveals a clearer dipole pattern, ranging from − 0.6 around 18°S to 0.7 north of the equator (Fig. 7c).

We have found a positive correlation (up to 0.4) between SLA and AMO particularly in the northern part of ETAO (i.e., from 4°S to 18° N, Fig. 7d). The AMO plays an important role in the North Atlantic circulation with less effect in the south^90,91^. Figure 7e depicts a high positive correlation (up to 0.6) between SLA and Nino3 index along the coasts from Nigeria to Gabon and mainly between 16°S and 24°S over the tropical South Atlantic, while the largest negative correlation (r = − 0.8) is observed in the equatorial region. This correlation suggests that the Nino3 index exerts a discernible influence on coastal sea levels, possibly through its effects on atmospheric circulation patterns and oceanic dynamics influenced by ENSO. During El Niño events (i.e. the positive phase of ENSO), coastal sea level rises significantly, while during La Niña events (i.e. the negative phase of ENSO), it falls significantly.

**Fig. 8:**
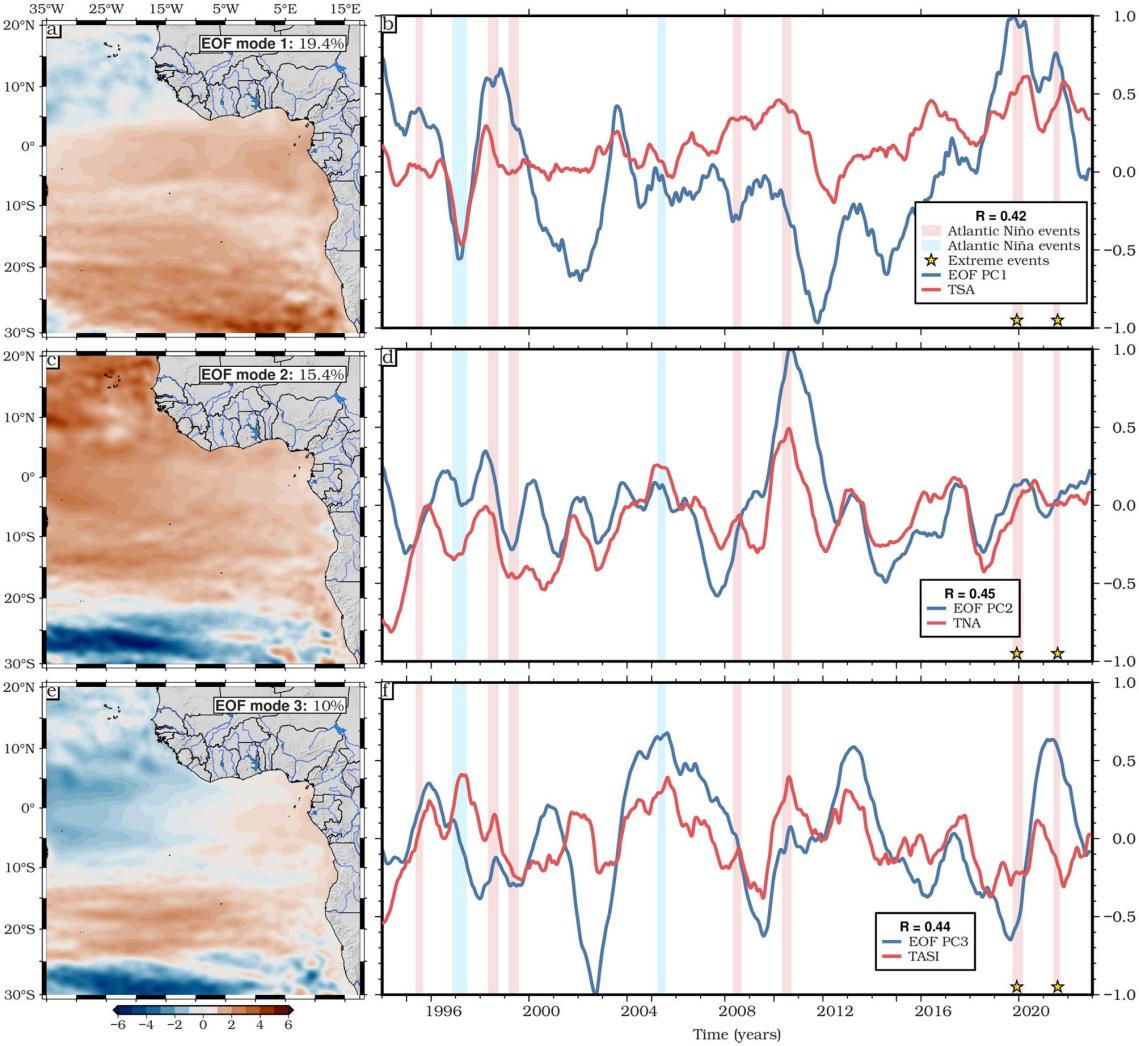
EOF decomposition of SLAs based on altimetry for the period 1993–2022. (**a**, **c**, **e**) Spatial patterns; (**b**, **d**, **f**) Principal components (PC). Time series of the TSA, TNA and TASI climate indices are shown together with modes 1, 2 and 3. R is the correlation coefficient between the PCs and the climate indices. For this EOF analysis the mean long-term trend was removed from all points to show the spatial patterns of the interannual changes.

The correlation between the Indian Ocean Dipole and the SLA (Fig. 7f) shows a strong positive correlation along the southern coastal region extending from GoG to the Angolan-Namibian freshwater system, with a strong positive correlation in the southern region, particularly along the southern subtropical gyre, suggesting that the DMI has a significant but latitude-dependent influence on the SLA. This result is consistent with the Empirical Orthogonal Function (EOF) analysis presented in Fig. 8, highlighting the dominant influence of the TSA, TNA, and TASI indices on SLA variability.

## Summary and conclusions

This study investigates the spatial and temporal trends of SLAs and their components (thermosteric and halosteric) in the ETAO from 1993 to 2022. The ETAO, home to the SMUS and NBUS, supports rich fisheries and is home to over 60 million people living within 10 m of sea level along the coast. However, this region faces the urgent threat of sea level rise due to climate change, putting coastal communities at risk of displacement and land loss. Our analysis reveals significant positive trends in overall SLAs across the ETAO, with pronounced trends observed along the coastal region of the GoG, where the trend exceeds 3.3 mm/year, indicating the region's vulnerability to sea level rise. A high SLA trend is also observed within the anticyclonic SASG. The thermosteric component contributes significantly to interannual sea level variability, closely following the detrended interannual variations in SLA. However, the overall SLA trend is predominantly explained by the manometric sea level changes. Conversely, the halosteric contribution is not statistically significant, with negative trends observed in some regions. Notably, the ocean mass component dominates the long-term sea level trend in the ETAO and its sub-basins, as confirmed by the GRACE and GRACE-FO missions from 2002 to 2022. This highlights the significant contribution of factors beyond thermal expansion and salinity changes, with a mean trend of 2.66 mm/year attributed to mass-induced sea level variations.

The ocean mass component is caused by the continued melting of glaciers and land ice on Antarctica and Greenland, though there may also be some changes in the output of the Congo, Volta, and Benue rivers. Additionally, changes in terrestrial water storage, including groundwater depletion and reservoir impoundment, may also contribute to the ocean mass component. The analysis further reveals significant interannual variability in SLAs, with notable peaks coinciding with major climate events such as the 1998, 2010, and 2021 Atlantic Niños and El Niño-Southern Oscillation (ENSO) events. The steric sea level component emerges as the main driver of this interannual SLA variability, potentially influenced by decadal oscillations and extreme climate events that can cause redistribution of water masses and changes in ocean circulation patterns, as noted by Forget and Ponte^78^. The sea level budget is nearly closed for the ETAO for the period April 2002 to December 2022, as the difference between the observed sea level and the sum of the steric and ocean mass components remains within uncertainty levels. The Tropical South Atlantic Index (TSA) emerges as a key driver of sea level variability in the region, with a strong correlation of 0.42 with the time series of the first EOF. Significant correlations (*p* < 0.05) are also observed between SLAs and other climate indices such as the Tropical Northern Atlantic Index (TNA), Tropical Atlantic SST Gradient Index (TASI), and Dipole Mode Index (DMI), highlighting the interconnectedness of regional sea level rise dynamics with broader climate phenomena.

These findings have profound implications for coastal communities and stakeholders in the ETAO region. Several of the relevant regional climate modes have persistence over timescales of many years, which, coupled with our revelations of their impact on coastal SLR, may enable government agencies to more reliably predict sea level changes affecting their citizens.

## Data and methods

### Satellite altimetry

Daily gridded SLA were extracted from the Copernicus Marine Environment Monitoring Service (CMEMS; 10.48670/moi-00148, accessed November 2023). This dataset covers the period from 1993 to 2022 with a horizontal spatial resolution of 1/8° × 1/8°. The monthly mean SLAs were calculated for each grid cell from the daily values. This product is a combination of all altimeter missions (Jason-3, Sentinel-3A, HY-2A, Saral/AltiKa, Cryosat-2, Jason- 2, Jason-1, T/P, ENVISAT, GFO, ERS1)^92^. This product was used to estimate the SLA trend and spatiotemporal SLA interannual variability in the ETAO during the above-mentioned period.

### Steric Sea Level Components

The ARMOR3D dataset (10.48670/moi-00052) is one of the most reliable products for the assessment of steric sea level fluctuations^93–96^. This product contains monthly temperature and salinity profiles obtained primarily from the Argo network using statistical methods with a horizontal resolution of 1/4° × 1/4° and 50 vertical levels (i.e., standard depths). The ARMOR3D dataset assimilates satellite (SLAs and surface geostrophic current components) and in-situ observations (temperature and salinity profiles). We then followed Jayne et al.^97^; Wang et al.^98^ and Mohamed and Skliris^99^ in using this dataset (over the same period as the altimetry) to estimate the SSLA, thermosteric anomaly (TSLA), and halosteric anomaly (HSLA) based on the following equation:

$$\begin{aligned} SSLA & = TSLA + HSLA = \frac{ - 1}{{\rho_{0} }}\mathop \smallint \limits_{ - H}^{0} \rho dz \\ & = - \mathop \smallint \limits_{ - H}^{0} \left( {\alpha \Delta T - \beta \Delta S} \right)dz \\ \end{aligned}$$

Here the reference density is ρ_0_ = 1025 kg/m^3^ and *z* refers to the depth. *Δρ*, *ΔS* and *ΔT* are the anomalies of density, salinity, and temperature, related to their baseline climatological mean (1993–2022) in each layer. The salinity contraction and thermal expansion coefficients, labelled β and α, were calculated using the Gibbs Sea Water (GSW) Oceanographic Toolbox and monthly temperature and salinity profile data, respectively, in accordance with the Thermodynamic Equation of Seawater-2010 (TEOS-10)^100^. *H* stands for the reference depth, which is set to 700 m or the seabed if shallower. According to the findings of Ghomsi et al.^19^, the steric effect is mainly limited to the uppermost 700 m.

### Atmospheric correction and trend analysis

Since the satellite altimetry was already atmospherically corrected, the DAC (V4.0), https://tds.aviso.altimetry.fr/thredds/catalog/dataset-auxiliary-dynamic-atmospheric-correction/catalog.html, accessed November 2023) was only used in our study to estimate the contribution of atmospheric forcing to SLAs variability. Linear trends of the SLA and steric components, as well as the residual sea level (total sea level minus steric), were estimated using the modified Mann–Kendall method with a 95% confidence interval^101^. Semi-annual and annual signals are removed by fitting 6- and 12-month period sinusoids to each time series. A thirteen-month running mean smoothing is further applied to all time series following Cazenave et al.^102^.

### GIA correction, ocean mass data analysis and spatial correlation computation for climate drivers

The relative sea level rise rate (dSea) and the geoid height change rate (dGeoid) from Peltier et al.^49^ are used to estimate the contribution of GIA correction to the altimetry data. We used gridded data from the GRACE mission provided by NASA's Goddard Space Flight Center (GSFC, https://earth.gsfc.nasa.gov/geo/%20data/grace-mascons). Assuming a water density of 1000 kg/m^3^, the data are available as equivalent sea level heights. This mass concentration solution is provided with a spatial resolution of 0.5° × 0.5° and a temporal resolution of one month, covering the period from April 2002 to December 2022, to ensure overlap with the satellite altimetry dataset. The ICE6G-D model^103,104^ was used to make corrections for the GIA effect. The modified Mann–Kendall test was applied to assess the significance of all our computations at the 95% confidence level^99^. In addition, spatial correlation analysis was conducted between SLA and the different climate modes listed in Table 1 to investigate the dominant drivers of climate variability in this region.

## Supplementary Information

Supplementary Information.

## Data Availability

All data supporting the results of this study is available in the paper.
