# Author Correction: Exploring steric sea level variability in the Eastern Tropical Atlantic Ocean: a three-decade study (1993–2022)

**DOI:** 10.1038/s41598-024-74673-1

**Published:** 2024-10-07

**Authors:** Franck Eitel Kemgang Ghomsi, Bayoumy Mohamed, Roshin P. Raj, Antonio Bonaduce, Babatunde J. Abiodun, Hazem Nagy, Graham D. Quartly, Ola M. Johannessen

**Affiliations:** 1 Deutsches Geodätisches Forschungsinstitut, Technische Universität München (DGFI-TUM), Munich, Germany; 2 Nansen-Tutu Center for Marine Environmental Research, Department of Oceanography, University of Cape Town, Cape Town, South Africa; 3 Geodesy Research Laboratory, National Institute of Cartography, P.O. Box 157, Yaoundé, Cameroon; 4 GeoHydrodynamics and Environment Research (GHER), University of Liège, Liège, Belgium; 5 Oceanography Department, Faculty of Science, Alexandria University, Alexandria, Egypt; 6 Nansen Environmental and Remote Sensing Center and Bjerknes Center for Climate Research, Bergen, Norway; 7 Marine Institute, Oranmore, Co.Galway, H91 R673 Ireland; 8 Plymouth Marine Laboratory, Plymouth, UK; 9 Nansen Scientific Society, Bergen, Norway

Correction to: *Scientific Reports*10.1038/s41598-024-70862-0, published online 03 September 2024

In the original version of this Article, the Acknowledgements section was incomplete.

"Franck Ghomsi is supported by the Nansen Scientific Society through the Nansen-Tutu Centre Fellowship Programme. R. P. Raj and A. Bonaduce are supported by the Sea Level Predictions and Reconstructions (SeaPR) project funded by the Bjerknes Center for Climate Research (BCCR) Strategic Projects Initiative. G. D. Quartly was supported by funding from the National Centre for Earth Observation (NCEO). The DRAGON 5 project of the European Space Agency is also acknowledged by the authors. We thank the editor, Bogdan Onac, and the two anonymous reviewers for their help in improving the final version of the manuscript. All plots were generated using Generic Mapping Tools (GMT), Version 6.5.0. https://www.generic-mapping-tools.org."

now reads

"Franck Ghomsi is supported by the Nansen Scientific Society through the Nansen-Tutu Centre Fellowship Programme. R. P. Raj and A. Bonaduce are supported by the Sea Level Predictions and Reconstructions (SeaPR) project funded by the Bjerknes Center for Climate Research (BCCR) Strategic Projects Initiative. G. D. Quartly was supported by funding from the National Centre for Earth Observation (NCEO). The DRAGON 5 project of the European Space Agency is also acknowledged by the authors. This project is supported by the National Research Foundation (NRF, South Africa). We thank the editor, Bogdan Onac, and the two anonymous reviewers for their help in improving the final version of the manuscript. All plots were generated using Generic Mapping Tools (GMT), Version 6.5.0. https://www.generic-mapping-tools.org."

The original Article has been corrected.

