# Supplementary material for: Exploring steric sea level variability in the Eastern Tropical Atlantic Ocean: a three-decade study (1993–2022)
Source: Sci Rep. 2024 Sep 3;14:20458. doi: 10.1038/s41598-024-70862-0 (PMC11371813; doi:10.1038/s41598-024-70862-0)
Supplement: Supplementary file 1 — Supplementary Information. [file 41598_2024_70862_MOESM1_ESM.docx]

**Supplementary materials**


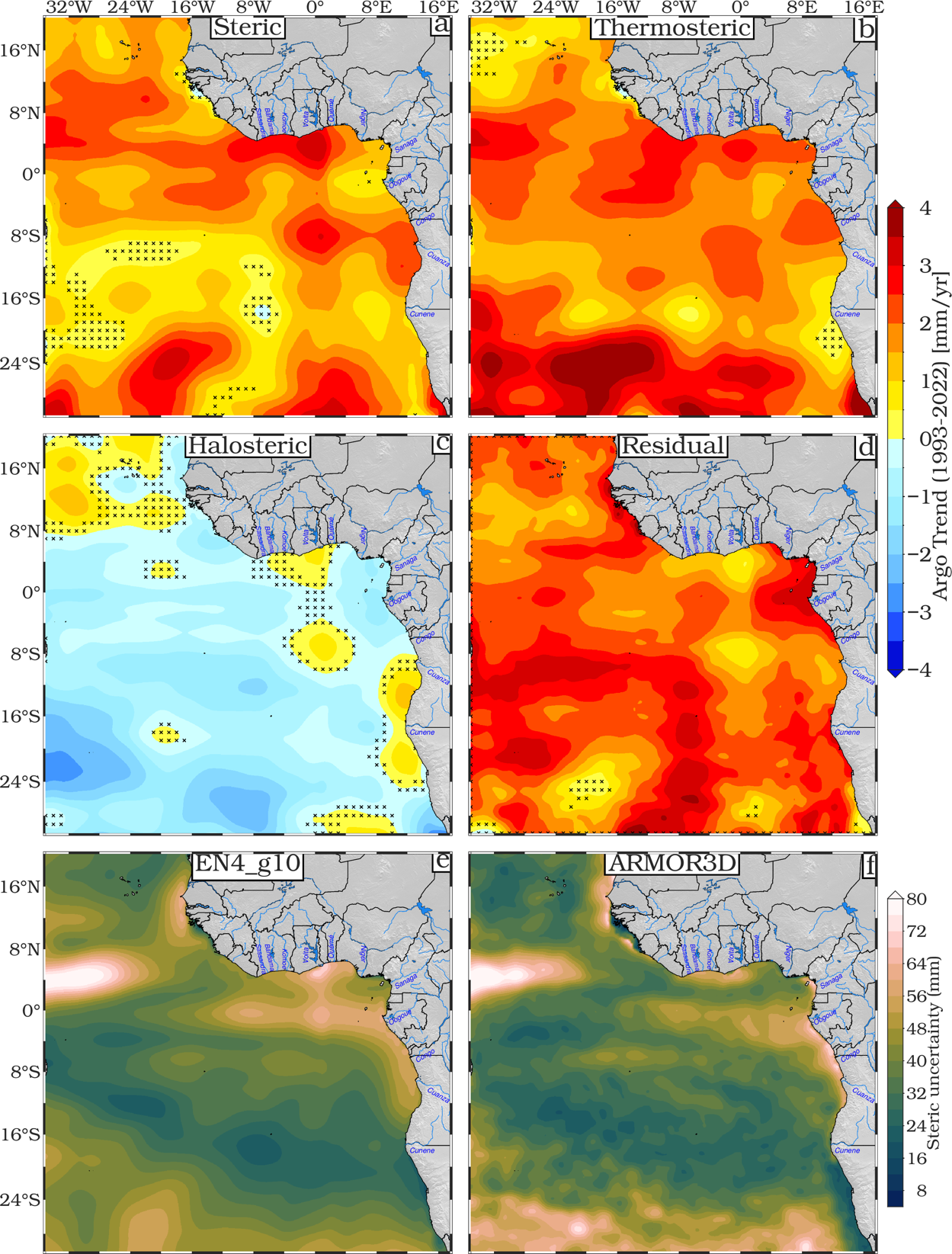
**Figure S1:** *EN4_g10 Argo-based spatial patterns of trends (mm/year) from 1993 to 2022 above 700 m after GIA correction for* ***(a)*** *steric effect,* ***(b)*** *thermosteric effect,* ***(c)*** *halosteric effect and* ***(d)*** *residual sea level. Mean and seasonal cycles have been removed from all time series at each grid point. Regions where trends are not statistically significant at the 95% confidence level are hatched. The uncertainties in the steric components are computed through the standard deviation for* ***(e)*** *EN4_g10 and* ***(f)*** *ARMOR3D.*

**Figure S2:** *De-seasoned monthly time series and linear trends (mm/yr) from EN4_g10 based solution for mean* ***(a)*** *SLA, steric, and residual (SLA minus steric) components and* ***(b)*** *steric, thermosteric, and halosteric sea level components over the ETAO for 1993-2022.*
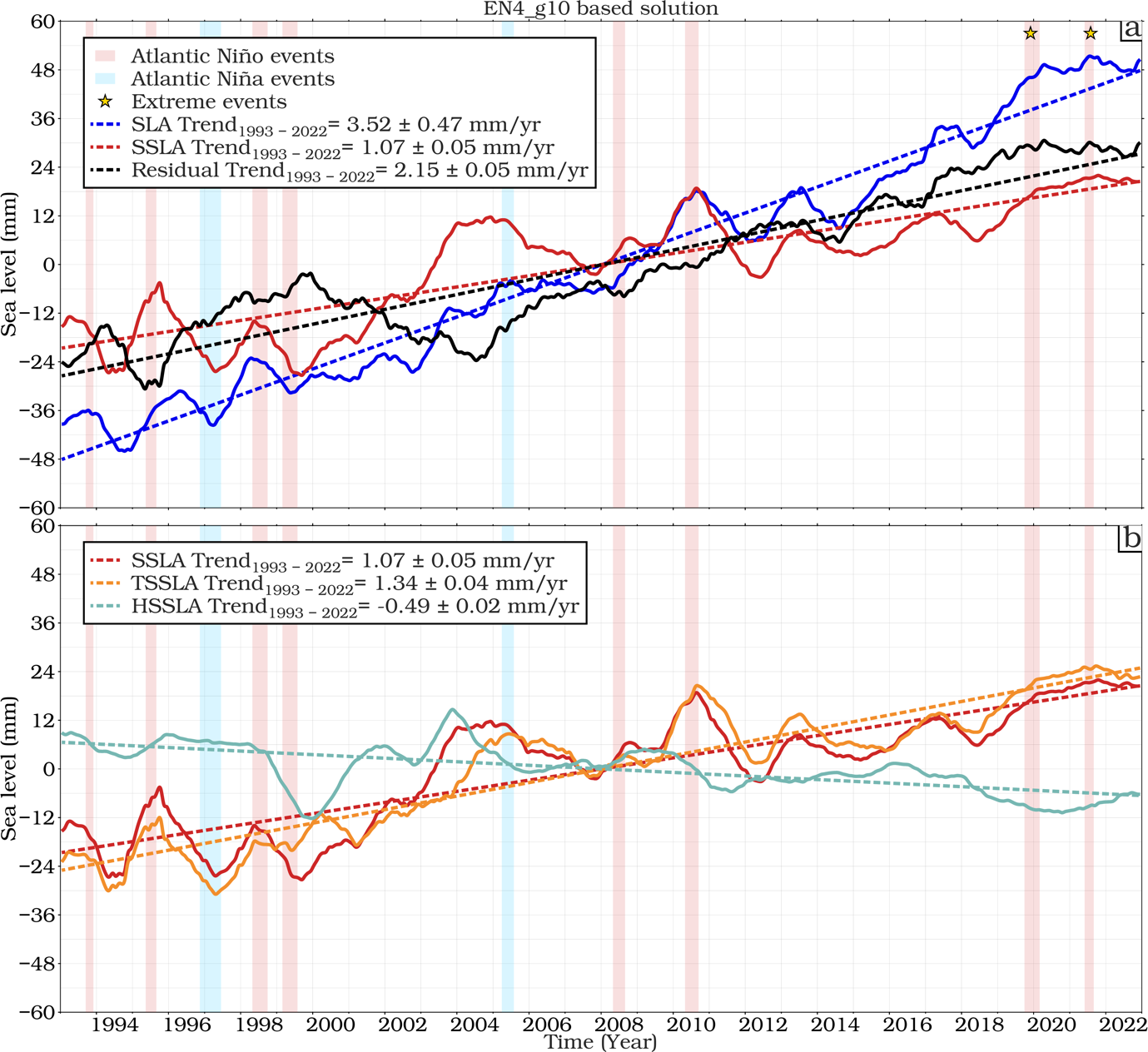


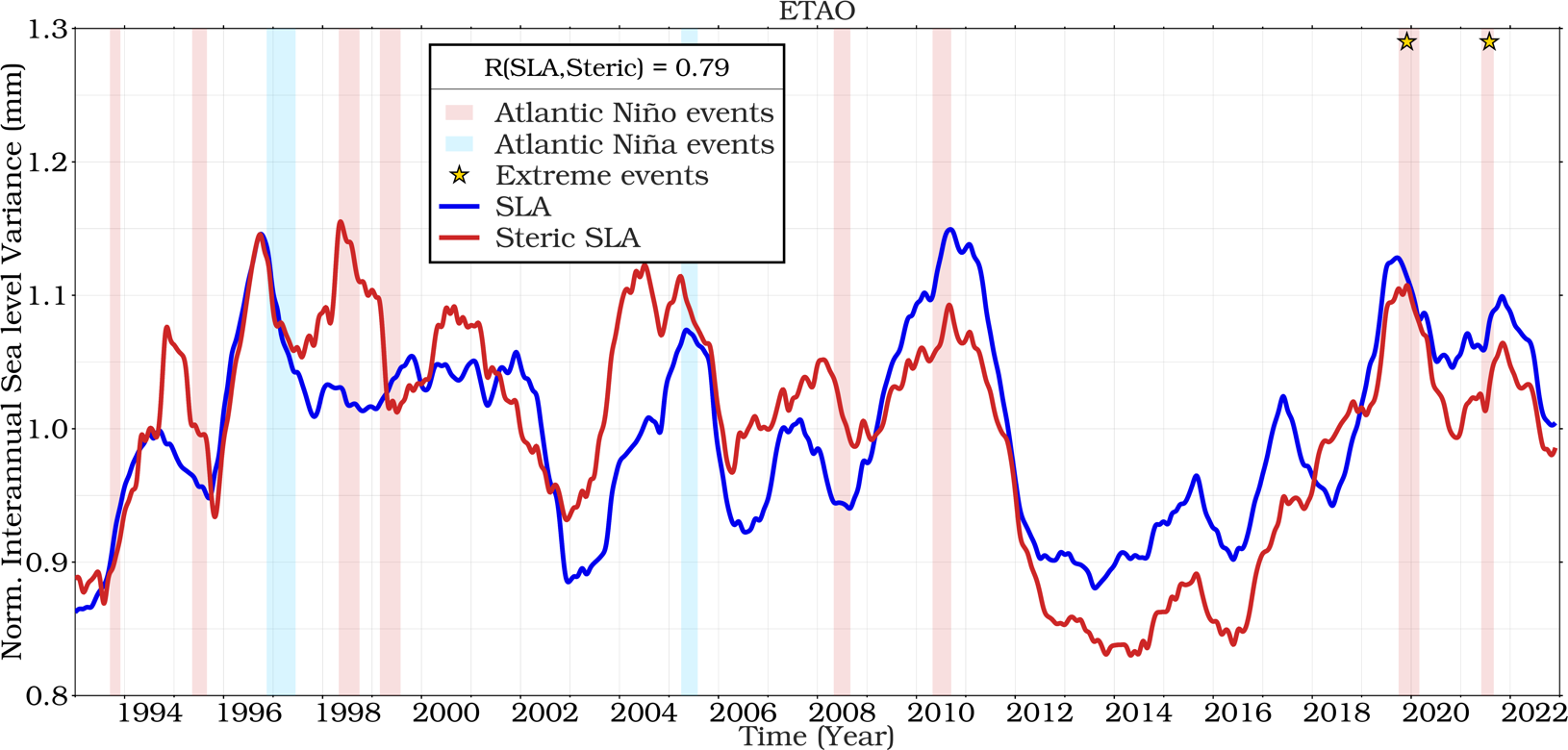


**Figure S3:** *Comparison of normalized Steric and Total Interannual sea level variance over the ETAO based on ARMOR3D.*
